# The Virology of Taterapox Virus In Vitro

**DOI:** 10.3390/v10090463

**Published:** 2018-08-29

**Authors:** Scott Parker, Leonardo Camilo de Oliveira, Elliot J. Lefkowitz, Robert Curtis Hendrickson, Cláudio A. Bonjardim, William S. M. Wold, Hollyce Hartzler, Ryan Crump, Robert Mark Buller

**Affiliations:** 1 Department of Molecular Microbiology and Immunology, Saint Louis University School of Medicine, 1100 South Grand Boulevard, St. Louis, MO 63104, USA; bill.wold@health.slu.edu (W.S.M.W.); hollyce@gmail.com (H.H.); rcrump1313@gmail.com (R.C.); 2 Grupo de Transdução de Sinal do Laboratório de Vírus, Departamento de Microbiologia, Instituto de Ciências Biológicas da Universidade Federal de Minas Gerais, Avenida Antônio Carlos 6627, Belo Horizonte, Brazil; deolive2@gmail.com (L.C.d.O.); bonjardim@icb.ufmg.br (C.A.B.); 3 Department of Microbiology, BBRB 276/11, University of Alabama at Birmingham, Birmingham, AL 35294, USA; ElliotL@uab.edu (E.J.L.); curtish@uab.edu (R.C.H.)

**Keywords:** orthopoxvirus, variola, ectromelia, smallpox, vaccinia, gerbil, taterapox

## Abstract

Taterapox virus (TATV) is phylogenetically the closest related virus to variola—the etiological agent of smallpox. Despite the similarity, few studies have evaluated the virus. In vivo, TATV can infect several animals but produces an inapparent infection in wild-type mice; however, TATV does cause morbidity and mortality in some immunocompromised strains. We employed in vitro techniques to compare TATV to ectromelia (ECTV) and vaccinia (VACV) viruses. Both ECTV and TATV replicate efficiently in primate cell lines but TATV replicates poorly in murine cells lines. Furthermore, TATV induces cytopathic effects, but to a lesser extent than ECTV, and changes cytoskeletal networks differently than both ECTV and VACV. Bioinformatic studies revealed differences in several immunomodulator open reading frames that could contribute to the reduced virulence of TATV, which were supported by in vitro cytokine assays.

## 1. Introduction

Variola virus (VARV) is the etiological agent of smallpox. Although smallpox no longer circulates in nature, significant concerns continue to exist that the virus could be reintroduced by nefarious mechanisms; furthermore, the prospect of genetically modifying VARV—or other orthopoxviruses (OPVs) such as monkeypox virus (MPXV)—is a real possibility that could render the host susceptible to disease even following vaccination and treatment with antivirals [1]. Taterapox virus (TATV) is phylogenetically the closest relative to VARV [2,3], so could therefore provide a platform for understanding and modelling the pathogenicity of VARV.

Until recently, only the original report describing the virus in 1975 had been published [4]. This paper by Lourie et al. revealed that a sole TATV isolate was recovered from an apparently healthy wild gerbil (*Tatera kempi* or *Gerbilliscus kempi*) captured in what is now the Republic of Benin. No other isolates have been reported. The assays performed in 1975 revealed that, when compared to other OPVs, TATV had many similar characteristics to VARV; however, in vivo assays were limited in number and description. In 2017, Parker et al. published a report that described TATV challenges in several small animals and found that the virus failed to induce significant morbidity in gerbils or wild-type mice but did induce seroconversion [5]. That said, immunocompromised *stat1*^−/−^ mice did experience morbidity, and death was experienced by severely combined immunodeficient (SCID) mice. Moreover, in some circumstances, mice were able to transmit TATV to cage-mates. These in vivo studies largely compared and contrasted TATV with ectromelia virus (ECTV) challenges. ECTV is the etiological agent of mousepox—a disease that is arguably the most suitable small-animal model of smallpox. The Parker et al. study suggested that ECTV provides a better platform for evaluating disease models, vaccines, and antivirals than provided by TATV challenges of small animals [5]. That being said, the paper did not report on the in vitro and bioinformatic characteristics of TATV; therefore, those studies are described in this report.

Here we reveal that several important open reading frames (ORFs) in the OPV’s compliment are truncated or are missing in TATV. Notably, ORFs involved in the perturbation of chemokines and cytokines were particularly affected, which typically resulted in cytokine responses that were radically different to those induced by ECTV. Further, the complement binding protein (CBP) found in many OPVs has been shown to be directly involved in virulence and is likely non-functional in TATV. In vitro replication studies revealed that TATV replicated poorly in mouse-derived cell lines and that plaque formation and morphology were highly-dependent on the host-cell type. Despite these differences, TATV appears to form Extracellular Enveloped Virions (EEV) in a normal fashion—similar to ECTV. Taken together, these data provide insights on why TATV replicates poorly in vivo in mice and is an inferior murine-based model of smallpox. Moreover, the studies reveal clues that could facilitate a better understanding of the factors involved in pathogenicity of other OPVs, both in vivo and in vitro.

## 2. Materials and Methods

### 2.1. Animals

The Institutional Animal Care and Use Committee at Saint Louis University School of Medicine approved all experimental protocols (protocol 2081, 15th July, 2012). BALB/c mice were acquired from Charles River Laboratories, (Wilmington, MA, USA).

All experimental and animal procedures were completed at ABSL-3, where animals were housed in filter-top microisolator cages. A standard rodent diet (Teklad Global 18% Protein Rodent Diet) and water were provided ad libitum. Corn cob bedding was provided in each cage, where no more than 5 animals were housed. 

### 2.2. Cells and Viruses

All cell lines, except I-10 and 1469, were grown in Eagle’s minimum essential medium (EMEM; Bio-Whittaker, Walkersville, MD, USA) containing 10% fetal bovine sera (FBS; Hyclone, Logan, UT, USA), 2 mM l-glutamine (GIBCO, Grand Island, NY, USA), 100 U/mL penicillin (GIBCO, Grand Island, NY, USA), and 100 μg/mL streptomycin (GIBCO, Grand Island, NY, USA). 1469 cells were grown in EMEM supplemented with 10% horse serum (ATCC, Manassas, VA, USA). I-10 cells were grown in F-12K media (ATCC, Manassas, VA, USA) supplemented with 2.5% FBS and 15% horse serum. Virus plaque assays were carried out on BS-C-1, CV-1, CHO, Pam 212, Vero, L929, RK13, A549, HeLa, and C57BL/6 MEF monolayers as previously described [6]. Viruses were propagated in BS-C-1 cells and purified through a sucrose cushion as described elsewhere [6]. Virus infectivity was calculated by titration on BS-C-1 monolayers for 7 days (TATV) or 4 days (ECTV) at 37 °C [7]. Plaques were visualised by addition of 0.5 mL 0.3% crystal violet/10% formalin to each well. Arithmetic means above the limit of detection (1 × 10^2^ PFU/mL) were calculated as PFU/g or PFU/mL [8].

To calculate virus growth curves, 0.01 PFU/cell of virus was adsorbed for 1 h on the indicated monolayers (BS-C-1, Vero, A549, L929, MEF, 1469, I-10, and C57BL/6 bone marrow-derived macrophages) at 37 °C. Cells were washed three times with phosphate buffered saline (PBS; 10% FBS *w*/*v*) and overlaid with the appropriate media. Cells were incubated and harvested at the indicated time points and the virus was titrated on BS-C-1 cells.

TATV was a gift from Geoffrey Smith (University of Cambridge, UK). The virus was isolated from a wild gerbil (*Tatera kempi*) caught in Dahomey, (now Republic of Benin) Africa in 1968 [4]. Plaque purified isolates of TATV, ECTV-MOS, and VACV-COP were propagated in BS-C-1 cells.

### 2.3. Cytoskeleton Dynamics

BS-C-1 cells (1 × 10^5^) were grown on 13 mm rounded coverslips and synchronically infected with either TATV, ECTV, or VACV at a multiplicity of infection (MOI) of 5. The infections were carried out at the times indicated in the figures, at which point the cells were fixed with 4% paraformaldehyde for 10 min. After fixation, the cells were permeabilized with PBS solution containing BSA 3% + Triton X-100 0.2% for 5 min followed by a 30 min incubation with PBS-bovine serum albumin (BSA) 3%-FBS 2% for blocking. Cells were then stained with specific primary antibodies: anti-β-tubulin (Millipore-Sigma, St. Louis, MO, USA) or anti-B5 (NR-428, BEI Resources, Manassas, VA, USA). Alexa 488-conjugated anti-mouse IgG was used as a secondary antibody (Molecular Probes, Thermofisher, Waltham, MA, USA) and DAPI (Millipore-Sigma) was employed to visualize cell nuclei and virus replication factories. To visualize actin cytoskeleton dynamics, cells were also labeled with Rhodamine-conjugated Phalloidin (Molecular Probes, Thermofisher). Fluorescently labeled cells were visualized using an Olympus FV1000 laser scanning confocal microscope at the Research Microscopy and Histology Core, Saint Louis University. Images were processed using ImageJ (NIH, https://imagej.nih.gov/ij/).

### 2.4. Cytokine Analysis

For cytokine assays, a bioplex cytokine assay kit (Bio-Rad, Hercules, CA, USA) was used to analyse 32 cytokines: IL-1a, IL-1b, IL-2, -3, -4, -5, -6, -7, -9, -10, -12p40, -12p70, -13, -15, -17, LIF, VEGF, MIG, LIX, MIP-2, IP-10, M-CSF, GCSF, GM-CSF, IFN-γ, eotaxin, KC, MCP-1, MIP-1a, MIP-1b, RANTES, and TNF-α. Assays were run on a Luminex-100 (Luminex, Austin, TX, USA) and analysed with xPONENT (Luminex, Austin, TX, USA) software (version 2.0). 

### 2.5. Microscopy

Light microscopy images were taken with a Zeiss (Oberkochen, Germany) dissecting microscope with a 3.2× objective lens. The images were captured using an Olympus (Shinjuku, Japan) 5.1 megapixel C-5060 wide zoom camera and were processed in Microsoft Power Point.

### 2.6. Bioinformatics

The comparative analysis of poxvirus genomes, ORF, and ORF fragments was carried out using the Poxvirus Genome Annotation System (PGAS) [9]. In brief, the ORF content of each genome was predicted on the basis of common orthologs in other poxvirus genomes, sequence similarity with proteins annotated in GenBank [10], conservation of known and predicted poxvirus promoter sequences located in the 5′ noncoding region upstream of the ORFs, and ORFs experimentally-determined to be expressed in either in vivo or in vitro systems. ORFs were characterized as intact, truncated, fragmented, or missing using a comparative genomics approach [9]. An ORF was labelled as intact if a predicted or experimentally determined promoter sequence was present at its 5′ end and if it was at least 80% or greater of the length of its intact orthologous counterparts. If an ORF was intact at the 5′ end and maintained a predicted promoter sequence, but that ORF would code for a protein that was less than 80% of the length of the intact orthopoxvirus protein, then it was labeled as a truncated ORF. Any ORF that lost its predicted promoter and/or had been significantly truncated at its 5′ end was categorized as a fragmented ORF. If no remnants of any significant sequence fragments could be detected for a particular gene, then that gene was annotated as missing in that virus. The accession numbers for each genome are: taterapox (TATV) (NC_008291), monkeypox (MPXV) (NC_003310), cowpox (CPXV) (DQ437593), vaccinia (VACV) (M35027), camelpox (CMPV) (AY009089), variola (VARV) (DQ441416), and ectromelia (ECTV) (NC_004105).

### 2.7. Statistics

Averages were calculated using the *t* test. *p* values below 0.05 were considered significant. All experiments were performed at least in triplicate.

## 3. Results

### 3.1. Comparison of TATV Genome with Genomes of CPXV, VARV, and ECTV

Phylogenetic analysis places TATV as the closest OPV relative to VARV [3,9] based on a comparison of nucleic acid sequences. TATV has a genome of approximately 196 kbp and encodes for 162 haploid and two diploid full length ORFs; 23 haploid and four diploid truncated ORFs; and six fragmented ORFs. The central conserved region (CCR) of OPVs comprises approximately 75% of the complete genome sequence, which is the most conserved region of the genome containing ORFs with high levels of orthology to other orthopoxviruses. We used the standard designation of the CCR as being between the orthologs of VACV-COP ORFs C7L and A51R. The ORFs encoded in the CCR typically code for proteins involved in ‘house-keeping’ activities, such as basic replicative processes, transcription, DNA replication, and virion assembly/packaging. ORFs located outside of the CCR are typically considered to be involved in virulence and host-range and are hypothesised to be tailored to the appropriate host(s) of each OPV.

CPXV is considered the most similar in ORF content to the common ancestor to all other known OPVs as it encodes the largest number of ORFs, and there are no ORFs that appear in any OPV species that are not also present in CPXV species [9]. Compared to CPXV, 25 ORFs are truncated, 6 are fragmented, and 18 are missing in the TATV genome (Supplemental Table S1). Compared to VARV, 13 ORFs are truncated, 2 are fragmented, and 2 are missing in the TATV genome (Supplemental Table S2). We also identified two VARV truncations that have fragmented orthologs in TATV, one VARV truncation that is missing in TATV, and five common VARV and TATV truncations (Supplemental Table S1 in Hendrickson et al., 2010 for a table of full, missing, fragmented, and truncated ORFs in orthopoxviruses [9]). The function of these common truncations cannot be determined; however, of these five common truncations, the highest level of truncation is always observed in the VARV ortholog, suggesting that the TATV truncations may be more, or equally as, functional as their respective VARV orthologs.

**Figure 1:**
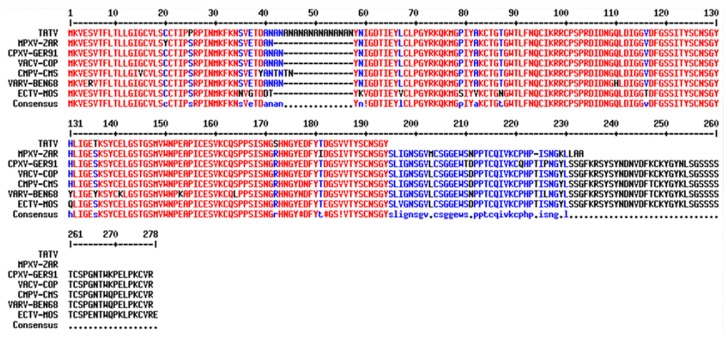
Alignment of the Complement Control Proteins from TATV, MPXV-ZAR, CPXV-GER91, VACV-COP, CMLV-CMS, VARV-BEN68, and ECTV-MOS. Regions of high consensus (90%) are shown in red; regions of low consensus (50%) are shown in blue.

To further dissect the genetic basis for virulence in TATV, we compared its genome to ECTV. We selected ECTV because it is a highly virulent natural mouse pathogen and the majority of our in vivo TATV studies were conducted in murine models [5]. Inspection of Table 1 reveals that 13 ECTV ORFs exist as truncations, 4 as fragments, and 8 are missing in the TATV genome. There are seven truncations in ECTV, of which three are also truncations in TATV, one is fragmented, and three are missing (Supplemental Table S1 in Hendrickson et al., 2010 [9]). Modification and perturbation of cytokines and chemokines is critical to ECTV virulence and we found two tumor necrosis factor receptor ORFs (crmD and vCD30), one IL-1 receptor agonist, one IL-1 signaling agonist, and one chemokine binding protein that were fragmented, missing, or truncated in TATV. Moreover, four ORFs encoding ankyrin repeats and the complement binding protein were also missing or truncated in TATV. The C3L complement binding protein (CBP) functions to inhibit the early steps of the host complement cascade. Disruption of the C3L ortholog reduces or alters the virulence of OPVs [11,12]. VARV, CPXV, VACV, camelpox (CMLV), and ECTV all encode a full-length version of C3L. OPV complement inhibitors consist of a series of four repeating complement control protein (CCP) domains. In MPXV isolates from Central Africa, the fourth CCP (CCP4) is truncated and the CBP ortholog in West African strains is completely missing. It is proposed that the absence of the CBP in West African strains plays a role in the reduced virulence levels of these viruses compared to Central African strains [11,13]. Despite the truncation in Central African strains, the protein still retains function equal to that of the VACV ortholog but less so than observed in the VARV ortholog. We compared the C3L ortholog of TATV to MPXV-ZAR (a Central African strain), CPXV, VACV, CMLV, VARV, and ECTV [14]. As shown in Figure 1, the TATV C3L ortholog has 84 residues deleted from the C-terminus of its protein compared to the full length C3L orthologs and 38 residues deleted from the C-terminus compared to the MPXV-ZAR C3L ortholog. Furthermore, inspection of Figure 1 reveals that residues 40–57 of the TATV C3L ortholog contain an extended alanine-asparagine repeat sequence that is 14 residues longer than that observed in CPXV, VACV, and VARV. In human CCP domains, the first cysteine residue typically pairs with the third cysteine residue and the second cysteine residue pairs with the fourth cysteine residue [15]. In TATV, the deletion removes the last two cysteine residues from CCP3. Thus, the TATV CBP is unlikely to have a properly folded CCP3. Furthermore, the extended alanine-threonine repeats in TATV may disrupt the folding of CCP1. Thus, it is likely that the absence of CCP4 and the potential misfolding of CCP3 and CCP1 will significantly reduce or completely ablate the activity of the TATV CBP.

### 3.2. In Vitro Replication Studies of TATV

**Figure 2:**
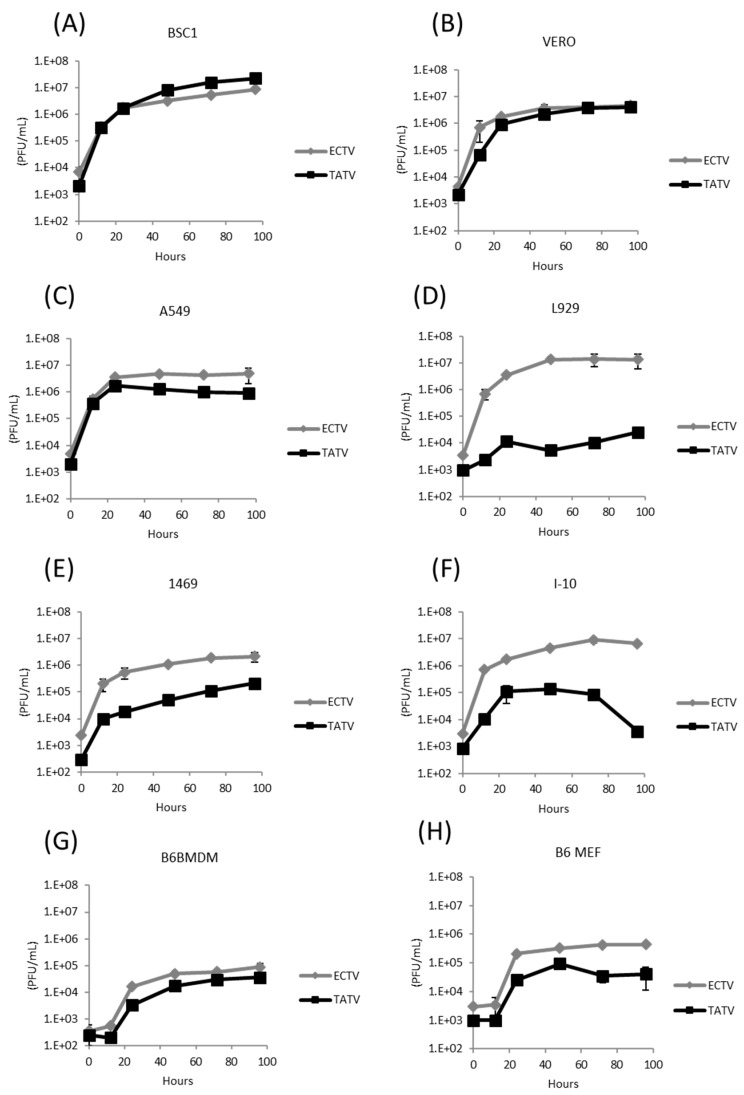
ECTV and TATV growth kinetics. Cell monolayers of (**A**) BS-C-1, (**B**) Vero, (**C**) A549, (**D**) L929, (**E**) 1469, (**F**) I-10, (**G**) B6BMDM, and (**H**) B6 MEF cells were infected with a multiplicity of infection (MOI) of 0.01 PFU/cell and total virus production was measured by plaque-assays on BS-C-1 cells at various times up to 96 hours post-infection (h.p.i.). Representative data of three experiments.

**Figure 3:**
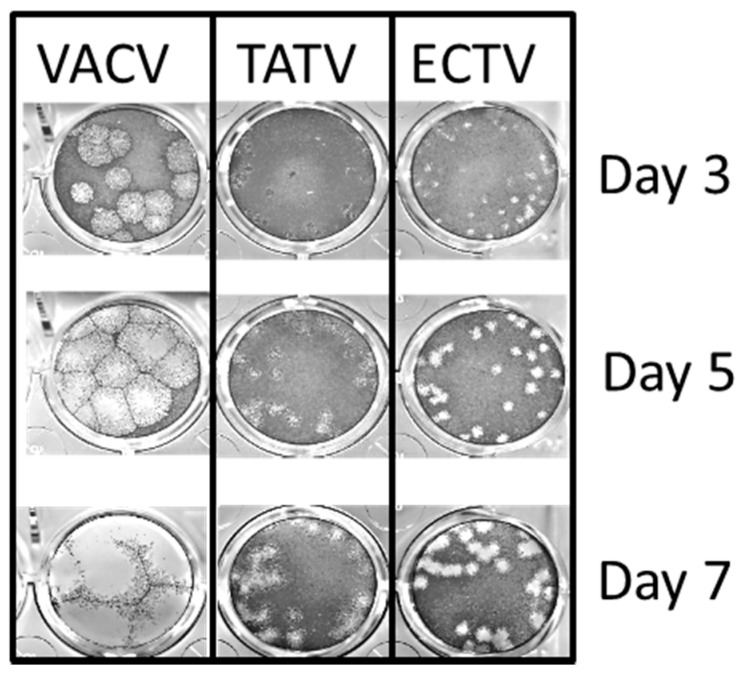
VACV, ECTV, and TATV plaque morphologies on BS-C-1 cells stained at day three, five, and seven days post-infection (p.i). Data are representative of three experiments. 1× magnification.

Compared to ECTV, TATV lacks a number of ORFs encoding proteins with ankyrin repeats. Ankyrin repeat-containing proteins, such as K1 and CHO, have been associated with the capacity to replicate in certain cell types (i.e., host-range) [16,17]. Accordingly, we compared replication of ECTV and TATV in BS-C-1 (*Cercopithecus aethiops*, epithelia), Vero (*Cercopithecus aethiops*, epithelia), A549 (*Homo sapiens*, epithelia, lung), L929 (*Mus musculus*, fibroblast), MEF (*Mus musculus*, C57BL/6 embryonic fibroblast), 1469 (*Mus musculus* fibroblast, liver), I-10 (*Mus musculus*, epithelial, testis), and C57BL/6 mouse bone-marrow derived macrophages (B6BMDM). As shown in Figure 2A–H, TATV and ECTV infectivity increased with similar kinetics when grown in BS-C-1, Vero, A549, and B6BMDM cells; although in A549 cells, TATV titers gradually decreased after 24 h. Compared to ECTV, TATV replicated with decreased efficiency in mouse 1469 and I-10 cell lines, and poorer still in the fibroblast cell line L929 and primary MEFs. In these cell lines, progeny virus production appeared reduced compared to ECTV. As expected from the BS-C-1 growth curve data, no qualitative or quantitative differences in TATV morphogenesis were observed by electron microscopy of infected BS-C-1 cells compared to ECTV. As shown in Figure 3, TATV plaque formation on BS-C-1 cells was distinct compared to VACV and ECTV. Clear VACV plaques were detected by day one post infection (p.i.) and could be easily counted by day two p.i. For ECTV infections, clear plaques were first detected on day three p.i. and these plaques could be counted on day four p.i. In comparison, for TATV, we could only detect pre-plaque CPE on day three p.i. The TATV plaques were far less discrete and the plaques were cloudy and the edges had a smudged appearance compared to plaques produced by ECTV or VACV. By day seven p.i., the TATV plaques were more distinct and could be easily counted; however, the plaques were less clearly defined than those of VACV and ECTV. Compared to BS-C-1 cells, TATV plaquing efficiency at day seven varied considerably among the cell lines (BS-C-1, CV-1, Vero, HeLa, A549, RK13, CHO, L929, PAM212, and MEF) and was highest in primate cell lines. In summary, TATV plaque formation and virus reproduction were highly cell-type- and species-specific with primate cells being more replication competent than mouse cells, except for mouse macrophages.

**Figure 4:**
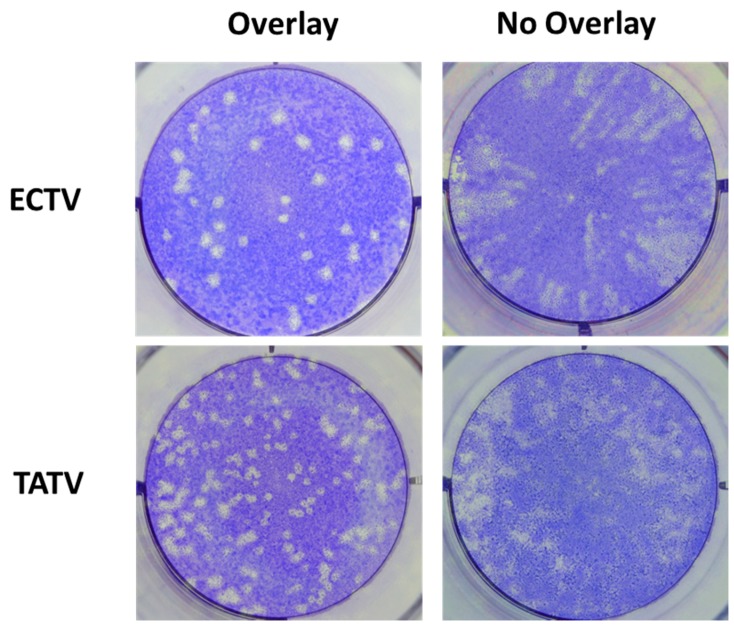
BS-C-1 cells were infected with ECTV or TATV. Wells were overlaid with CMC to reveal standard plaques or were not overlaid with CMC to reveal comet-plaques. Data are representative of three experiments. 2× magnification.

One explanation for the less defined or cloudy plaques is the less efficient cell-to-cell spread of virus and/or the reduction in extracellular enveloped virus (EEV). To qualitatively measure EEV production, we plaqued the virus in the absence of CMC overlay media [7]; this technique removes the restriction of EEV movement in the media allowing the formation of comet-shaped plaques [18]. We used a comet assay to compare TATV with ECTV and found that ECTV formed typical comet-shaped plaques indicating production of EEV; however, TATV did not form typical and easy-to-identify comet-shaped plaques (Figure 4). The failure of comet plaque formation could be due to reduced levels of EEV, failure of EEV to leave the infected cell, or to the inherently unconventional plaque morphologies formed on BS-C-1 cells by TATV. 

### 3.3. In Vitro Cytoskeletal Network Changes Following Infection

To further dissect the in vitro dynamics of TATV, we compared cytoskeletal changes in BS-C-1 cells infected with TATV, ECTV, and VACV. Cells infected with VACV undergo dramatic changes in both microtubule (MT) and actin cytoskeleton architectures, leading to cellular alterations collectively known as Cytopathic Effects (CPE) [19,20]. These sets of alterations are induced throughout the VACV replication cycle in order to enhance both MT and actin-based VACV motility [20,21]. Therefore, virus replication and efficient dissemination of wrapped particles is dependent on a precise regulation of host cytoskeleton networks [18,20,21]. Since the plaques formed by both TATV and ECTV are significantly reduced in size compared to VACV (Figure 3), we sought to analyse the changes induced by TATV and ECTV in the host cytoskeleton architecture during infection, in comparison with VACV.

**Figure 5:**
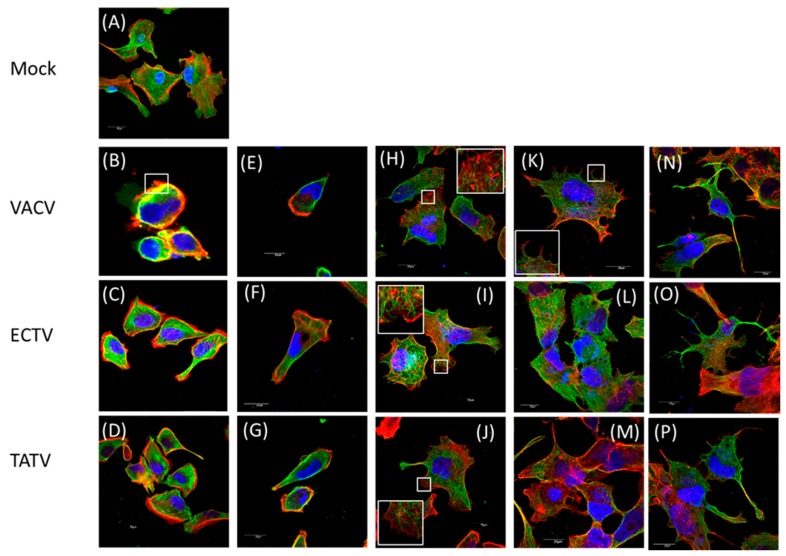
Detection of microtubules and actin filaments by confocal microscopy. BS-C-1 cells were infected at an MOI of five (or mock, **A**) with either (**B**,**E**,**H**,**K**,**N**) VACV, (**C**,**F**,**I**,**L**,**O**) ECTV, or (**D**,**G**,**J**,**M**,**P**) TATV. After 4, 8, 12, 24, or 30 h.p.i., the cells were fixed, permeabilized, and stained. Nuclei (blue), F-actin (red), and microtubules (green) are shown. (**A**) Representative image of uninfected cells. (**B**–**D**) Infected cells at four h.p.i.; (**B**) VACV cells are displaying the retracted, rounded CPE-M 1. (**E**) Migratory CPE-M 2 induced after VACV infection (**H**) at four h.p.i. Re-flattened, stretched CPE-M 3 observed at 12 h.p.i. with VACV. Inset depicts the virus-propelling actin tails displayed all over the infected cell body. (**K**) Pre-terminal stage of VACV infection. The CPE-M 4 represents the onset of cell death. Inset depicts the formation of projections at the cell periphery. (**N**) End-stage of VACV infection is observed here and represented by CPE-M 5. The infected cell displays a multi-branched morphology, with several protrusions formed by both actin and microtubule filaments. This is the terminal stage of VACV infection and CPE-M 5 evolves to cell death. TATV and ECTV cells are shown for comparison. 1000x magnification.

**Figure 6:**
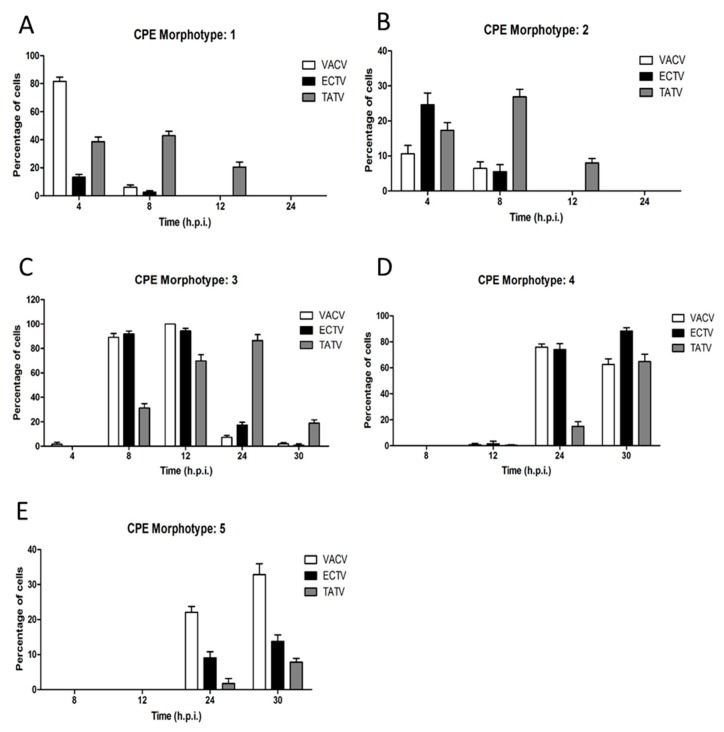
The occurrence of CPE morphotypes one to five in VACV-, ECTV-, and TATV-infected BS-C-1 cells. Cells were infected and prepared as outlined in the legend of Figure 5. The percentage of cells with (**A**–**E**) morphotype 1–5, respectively, were reported at 4, 8, 12, 24, and 30 h.p.i.

Infection of cells with orthopoxviruses induce the appearance of distinct cytophatic effect-associated cell morphotypes (CPE-M) [20]. In our analysis, we qualitatively categorized each CPE-M from one to five (Figure 5) with respect to the time course in which they appear [19,20]. Further, we employed morphometric analysis to determine both the prevalence and abundance of each CPE-M induced during the time-course of infection (Figure 6).

**Figure 7:**
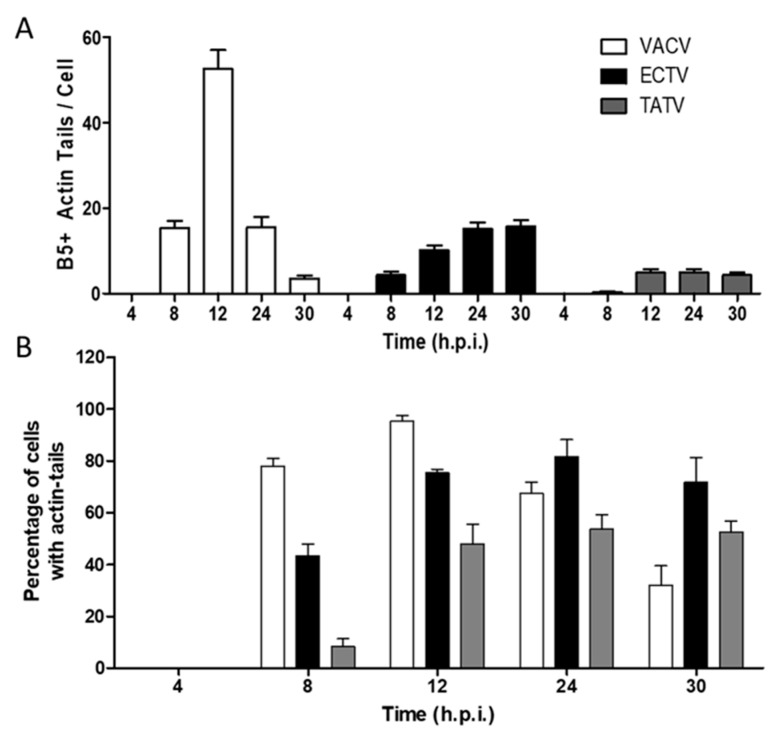
BS-C-1 cells were infected at an MOI of five, as described previously. After 4, 8, 12, 24, and 30 h.p.i., cells were fixed and stained with Rhodamine-conjugated Phalloidin to mark the actin cytoskeleton, as well as with the anti-VACV B5 envelope protein. B5-positively marked actin tail accumulation over the course of infection with the different OPVs, VACV, TATV, or ECTV, was quantified and plotted. (**A**) The average number of actin tails per cell during infection. (**B**) The percentage of cells displaying at least one B5-positve actin tail in the cell body was determined during the time-course of infection with VACV, TATV, or ECTV. Graphs were plotted with Graphpad Prism 6.0. Data are representative of three experimental replications.

VACV-induced CPE is well described, and the early phase is characterised by a cell rounding morphotype (CPE-M 1; Figure 5B) that predominates at four hours post infection (h.p.i.). In ECTV infected BS-C-1 cells, CPE-M 1 predominated at four h.p.i. (Figure 5C) with a relative abundance significantly smaller than what was observed for VACV (~82% of the infected cells). CPE-M 1 predominated until 12 h.p.i. in TATV infected cells (Figure 5D), whereas in VACV- and ECTV-infected cells, CPE-M 1 was present only until eight h.p.i. (Figure 7).

VACV-infected cells undergo, through migration, an event that relies on early viral gene expression [22,23] and repression of RhoA signaling by the viral protein, F11L [23]. Typical migrating cells (categorized as CPE-M 2; Figure 5E–G) were observed in cells infected with TATV and ECTV, as well as with VACV. In our analysis, CPE-M 2 appeared in VACV-infected BS-C-1 cells in the intervals of four and eight h.p.i. (~11% and 6% of infected cells, respectively). CPE-M 2 was present during infection with ECTV (Figure 5F) with similar kinetics as in VACV, even though ECTV-induced migration at four h.p.i. presented in higher abundance in comparison with VACV (Figure 6). Interestingly, CPE-M 2 appearance in BS-C-1 cells infected with TATV (Figure 5G) was significantly more frequent. We were able to observe the presence of CPE-M 2 until 12 h.p.i. in TATV-infected cells. Furthermore, relative abundance of CPE-M 2 at eight h.p.i. was around 27% for TATV-infected cells, compared to approximately 6% for both ECTV and VACV, suggesting that the migration event induced by TATV is more frequent in comparison with ECTV and VACV (Figure 6).

By eight h.p.i., VACV-infected cells flattened and MTs protruded from the center toward the cell periphery. This morphotype was scored as CPE-M 3 and predominated in the intervals of 8 up to 12 h.p.i. in VACV-infected cells (Figure 5H and Figure 6). CPE-M 3 is the main morphotype associated with cell-to-cell dissemination, and the one where the distribution of virus-propelling actin-tails is more robust. In ECTV-infected BS-C-1, CPE-M 3 (Figure 5I) predominated in the same intervals as observed for VACV (Figure 5H and Figure 6). However, abundance of virus-propelling actin-tails in ECTV-infected cells was significantly reduced in comparison with VACV (Figure 7A). Further, ECTV-infected cells presented with actin tails until the last time point analyzed in this study. The peak of actin tail accumulation in ECTV-infected cells was reached after 24 h.p.i. and remained steady until 30 h.p.i., whereas in VACV-infected BS-C-1, the relative abundance of actin tails reached the maximum accumulation at 12 h.p.i. (Figure 7A). Interestingly, TATV-infected BS-C-1 cells evolve to CPE-CM 3 (Figure 5J) stage of infection even though cells presented with significantly reduced levels of actin-tail accumulation when compared to both VACV and ECTV (Figure 7A). Furthermore, we observed that only 50% of TATV-infected BS-C-1 cells presented with actin-tails in the intervals of 12, 24, and 30 h.p.i. (Figure 7B). Notably, wrapped virus-propelling actin-tail, in all three orthopoxviruses analyzed here, presented with similar morphology and size.

CPE-M 3 is replaced by multi-branched cells with several protrusions in VACV-infected cells (Figure 5K). This morphotype was scored as CPE-M 4 and represents the onset of transition to terminal stages of VACV infection. CPE-M 4 predominated at 24 and 30 h.p.i. for all viruses (Figure 6). The kinetics of CPE-M 4 appearance in ECTV-infected BS-C-1 was similar to VACV (Figure 5K,L), though we observed an increase in the predominance of CPE-CM 4 at 30 h.p.i.; whereas in VACV, the proportion of CPE-M 4 decreased at 24 h.p.i. In contrast, CPE-M 4 appeared in a reduced proportion after 24 h.p.i. (~15% of infected cells) in TATV-infected cells (Figure 5M and Figure 6) in comparison with both VACV and ECTV.

The terminal morphotype was scored as CPE-M 5 and represents the ending stage of VACV-infection, in which the host-cells evolve to cell death. By 24 h.p.i., approximately 22% of BS-C-1 cells infected with VACV presented with CPE-M 5—a proportion that increased to approximately 33% by 30 h.p.i. Interestingly, the appearance of CPE-M 5 seems to be delayed in BS-C-1 cells infected with TATV (Figure 5P) or ECTV (Figure 5O), in comparison with VACV (Figure 5N). By 24 h.p.i., abundance of CPE-M 5 was 9% and 2% for ECTV and TATV-infected cells, respectively. After 30 h.p.i., CPE-M 5 abundance was approximately 13% and 8% in ECTV and TATV-infected cells, respectively (Figure 6).

### 3.4. In Vitro Cytokine Changes in Splenocytes Infected with ECTV or TATV

In the 2017 study published by Parker et al., they demonstrated that TATV inoculations by the FP route failed to broadly activate cytokine synthesis in the PLN [5]. To expand on this, we wanted to directly evaluate TATV’s capacity to induce cytokine responses from lymphoid cells. To this end, splenocytes from BALB/c mice were infected in vitro with an MOI of 0.1 for TATV or ECTV, and supernatants were assayed at 12, 24, and 48 h.p.i. for cytokines. The following cytokines were not significantly changed in BALB/c splenocytes infected with ECTV or TATV: Eotaxin, GM-CSF, LIF, LIX, IP-10, IL-1a, -1b, -2, -3, -4, -5, -6, -7, -9, -10, -12p40, -12p70, -13, -15, and -17 (data not shown). Significant changes were observed in all other tested cytokines compared to mock-infected controls (Table 2). Growth factor KC, VEGF, and M-CSF levels increased in TATV and ECTV-infected splenocytes, with maximal levels of KC observed in TATV infections, whereas VEGF and M-CSF concentrations were higher in ECTV infections. TNF-α levels were similar in TATV and ECTV infections until 48 h.p.i. when the TNF-α concentration was ca. two-fold higher in TATV infections. A clear pattern was observed with all measured chemokines. Except for MIG and KC, chemokines were virtually undetectable following ECTV infection, whereas significant quantities were detected in TATV supernatants. The higher chemokine levels in TATV infections could not be explained by increased robust replication of TATV as the ECTV titers in splenocyte cultures were ca. one log higher than TATV at 24 and 48 h.p.i. Thus, the in vitro chemokine response of splenocytes infected with TATV and ECTV appeared to be strikingly different.

## 4. Discussion

TATV was first isolated in 1975, and until recently, no other studies have been published on its virology. Our previous paper [5] evaluated TATV in several small animals and found that it cannot initiate robust disease in immunocompetent mice, the *Mongolian gerbil* (*M. unguiculatus*), or the *African dormouse* (G. Kelleni). However, as measured by seroconversion, we were able to determine that TATV infected mice and the Mongolian gerbil. Immunocompromised stat1^−/−^ mice challenged via the intranasal or footpad routes with TATV experienced morbidity but did not experience mortality. However, SCID mice did eventually die after a protracted disease course of more than 35 days. These findings support data presented here that suggest that TATV has a very narrow host range and is highly attenuated. Therefore, we surmise that to fully evaluate the virology of TATV in vivo and in vitro, the natural host of TATV would need to be identified. Given that the natural host of many OPVs—such as VACV—have yet to be identified despite a plethora of publications and studies, the likelihood of identifying the natural host of TATV in the near future is small.

In general, we found that both ECTV and VACV had clear temporal demarcations between CPE M types 1 to 3; this was typified by a rapid increase in cells presenting with the respective morphotype followed generally by a rapid decrease and entry into the subsequent CPE morphotype. For example, CPE-M 1, CPE-M 2, and CPE-M 3 were typically discrete events that could be clearly demarked temporally. However, TATV CPE-M 1 to 3 was a more protracted event lasting significantly longer and taking a longer time to reach peak levels. These findings could indicate that TATV-infected cells are more resilient in retarding the transition from CPE-M 1 to 3. In ECTV, CPE-M 4 and 5 began earlier, reached very high levels, and continued to increase beyond the 30 h assay window. However, in TATV-infected cells, the increase was delayed and more protracted, again indicating that the cells could be more resilient to TATV compared to ECTV. Taken together, our findings suggest that TATV is infecting the cells less efficiently than ECTV, which is possibly why plaques were cloudy. A possible reason for this finding could be related to the fact that a maximum of ~60% of TATV-infected cells were present with actin tails compared with ~100% and ~80% in VACV and ECTV, respectively. Furthermore, there are far fewer actin-tails/cell in TATV-infected cells (~5 tails/cell) compared to VACV (~50 tails/cell) and ECTV (~20 tails/cell). One possible conclusion could be that that EEV is being trapped within the cell and is not able to engage in long-range transmission—a finding that is supported by the failure of TATV to develop comet-plaques but to replicate fairly robustly when measured using growth-curves.

Many TATV ORFs were identified that were truncated, missing, or fragmented with respect to other OPVs. One likely candidate that may be responsible for the attenuated nature of TATV is the CBP, which is likely non-functional. All other OPVs examined had functional CBPs. In the case of MPXV, the West African strains completely lack the CBP and are significantly less virulent than the Central African strains, which retain a truncated CBP that is still functional [11]. An important experiment that could be considered would be to replace the TATV CBP with that of another OPV and to screen for increased virulence.

A46R is an ORF found in all examined OPVs but is truncated in TATV. In VACV, A46R likely plays a role in determining disease outcomes in mice [24] and VACV has been shown to be attenuated in an IN mouse model when the A46R gene was removed [24]. The protein has been shown to inhibit IL-1 signaling through interaction with Toll/IL-1 receptor domain-containing proteins Mal, TRAM, MyD88, and TRIF, which results in the inhibition of TLR NF-kB, MAPK, and IRF3 activation, and also blocks RANTES and IL-8 [25]. These findings suggest that A46R plays a role in immune evasion in OPVs. Assuming A46R plays a similar role in ECTV, then these data support our findings that ECTV can, but TATV cannot, reduce the level of RANTES in infected PLNs (Table 2).

The role of the TNF receptor (TNFR) ORFs is clearly important to the OPV host-range. CPXV, which has the broadest host-range, encodes up to five of these ORFs (vCD30, crmB, crmC, crmD, and crmE (Table 3). OPVs, except for TATV and VACV, encode at least one of these five ORFs. The reason for the variety in TNFRs encoded by OPVs is not fully understood and does not appear to be related to host-range. crmB binds a restricted set of chemokines through its C-terminal SECRET (smallpox virus-encoded chemokine receptor) domain [26]. This domain is also found in crmD, another TNFR ortholog that binds TNF-α and TNF-β, and is found in CPXV and ECTV [26,27]. Although TATV is missing crmB and crmD, it does encode at least two proteins containing SECRET domains and four proteins that belong to the 35 kD major secreted virus protein family that bind both CC and CXC chemokines [28], which likely imbue TATV with some chemokine binding activity. Except for TATV and VACV, which likely have non-functional crmB orthologs [29], it appears that OPVs that encode crmB can afford to lose the four other TNFR ORFs and maintain virulence. Conspicuously, ECTV is missing crmB but encodes vCD30 and crmD. vCD30 is a secreted protein shown to block IFN-γ producing cells, found in CPXV and ECTV but not in TATV, CMLV, VARV, or VACV [30]. The TNF-α receptor homolog, crmE, appears to have redundancy because it is only encoded by some CPXV species and is either missing of truncated in other examined OPVs. However, crmE has been shown to play a role in virulence in VACV-USSR [31,32]. The TNF-α receptor A53R ortholog, crmC, also appears to have redundancy because it is encoded only by CPXV and is predicted to be a non-functional truncation in VACV-COP and WR. However, a functional copy of crmC is found in a few VACV strains such as Lister [29,33]. Thus, it appears that only three of the five TNFR genes are critically important for virulence, and of these three, an OPV must encode as a minimum, either crmB or both vCD30 and crmD. The only exception to this rule is VACV and TATV, which likely have non-functional crmBs (due to truncations), which may explain their low levels of virulence in most models [29,34].

B29R/C23 is a chemokine-binding protein (vCCI) that has been shown to bind a wide variety of different chemokines. In particular, it has affinity for CC chemokines that play a role in the pro-inflammatory response and has been shown to act as an anti-inflammatory agent in vivo [35,36]. vCCI is truncated by 74% in TATV but is a full protein or a protein truncated by ≤5% in all other OPVs examined. vCCI has been shown to inhibit the pro-inflammatory cytokines MIP-1α and MIP-1β [37,38]. These findings are consistent with our data (Table 2), which show that MIP-1α and MIP-1β levels are almost totally abolished in splenocytes infected with ECTV, but remain high in splenocytes infected with TATV. Our data strongly support the hypothesis that the inflammatory response is diminished by ECTV but not TATV in vitro and in vivo (Table 2) [5]. In addition to MIP-1α and MIP-1β, we found several other cytokines, including MIP-2, KC, MCP-1, and RANTES, that were significantly lower in ECTV-infected splenocyte cultures compared to TATV-infected splenocyte cultures. Reduced levels of MCP-1 and RANTES in ECTV-infected splenocytes is not surprising as both are inhibited by the vCCI. Therefore, an absent vCCI in TATV would be expected to result in an enhanced inflammatory response at the primary site of virus replication in the skin, and would contribute to restricted replication and systemic spread.

In summary, we found that the replication kinetics of TATV are significantly different to those of VACV and ECTV. We also identified several non-functional ORFs that could be responsible or co-responsible for the apparent reduced virulence level of TATV both in vivo and in vitro.

## Figures and Tables

**Table 1:** Intact open reading frames (ORFs) found in ectromelia virus (ECTV) that are fragmented, missing, or truncated in taterapox virus ^1^ (TATV).

			TATV		ECTV	
CPXV-GRI/GER Stop ^2,3^	VACV-COP Ortholog (CPXV Protein Size)	Function (Motif)	Status	Protein Size (% Compared to CPXV)	Status	Protein Size (% Compared to CPXV)
35,634	C2L (512)	Unknown (Kelch-like)	Frag ^4^		ORF018	512 (100)
38,413	N2L (175)	Alpha amanitin sensitivity protein	Frag		ORF020	177 (101)
170,420	A46R (240)	Interleukin 1 (IL-1) signalling inhibitor	Frag		ORF145	240 (100)
191,696	B12R (283)	Serine/Threonine kinase	Frag		ORF152	286 (101)
9217	(273)	Unknown	Miss		ORF004	273 (100)
10,160	(655)	N-term ankyrin, C-term F-box	Miss		ORF005	650 (99)
12,346	(96)	C-type lectin	Miss		ORF006	103 (107)
14,342	(202)	Unknown (partial homology to TNFR CrmB)	Miss		ORF008	202 (100)
14,947	(111)	Tumour necrosis factor receptor (vCD30)	Miss		ORF009	111 (100)
15,374	(764)	Unknown (Ankyrin)	Miss		ORF010	763 (100)
32,306	(29)	Unknown	Miss		ORF ^7^	29 (100)
213,858	(322)	Tumour necrosis factor receptor (CrmD)	Miss		ORF003/170	320 (99)
23,924	C10L (331)	Interleukin 1 (IL-1) receptor antagonist	Trnc ^5^	27 (8)	ORF011	331 (100)
34,788	C3L (259)	Complement binding protein (secreted)	Trnc	194 (75)	ORF017	262 (101)
41,155	K1L (284)	NFkB inhibitor (Ankyrin)	Trnc	88 (31)	ORF022	285 (100)
73,579	O1L (666)	Unknown	Trnc	64 (10)	ORF052	666 (100)
150,930	A25L (1279)	A-type inclusion protein	Trnc	736 (58)	ORF128	1113 (87)
165,419	A39R (402)	Semaphorin	Trnc	134 (33)	ORF139	399 (99)
168,244	A44L (346)	Hydroxysteroid dehydrogenase	Trnc	88 (25)	ORF143	346 (100)
169,708	A45R (125)	Superoxide dismutase (Cu-Zn) like protein	Trnc	46 (37)	ORF144	125 (100)
171,963	A48R (204)	Thymidylate kinase	Trnc	58 (28)	ORF147	204 (100)
178,726	A55R (564)	Kelch-like	Trnc	219 (39)	ORF150	563 (100)
197,359	B18R (574)	N-term ankyrin, C-term F-box	Trnc	147 (26)	ORF165/010	574 (100)
222,274	B29R/C23 ^6^ (255)	Chemokine binding protein	Trnc	61 (24)	ORF001/172	247 (97)
172,501	A49R (162)	Phosphotransferase anion transport protein (putative)	Trnc	77 (48)	Trnc	116 (72)

^1^ All intact, missing (Miss), truncated (Trnc), or fragmented (Frag) ORFs common between TATV and ECTV are removed. All truncated ECTV ORFs that are full-length ORFs in TATV are removed. ^2^ The stop codon is used for ORF identification as there sometimes can be multiple in-frame ATG’s located proximal to the 5′ end of the transcript, complicating the identification of the precise start codon of the protein. ^3^ Orthopoxvirus central conserved region exists between cowpox virus (CPXV)-GRI ORF Host Range Virulence Factor (stop codon 31795, VACV-COP C7L) and an ORF of unknown function (stop codon 175249, VACV-COP A51R) which corresponds to approximately 15.3–157.3 kbp in TATV and 24.2–167.2 kbp in ECTV. ^4^ Any ORF that has lost its predicted promoter and/or has been significantly truncated at its 5′ end is annotated as a fragmented gene. ^5^ ORFs that maintain a predicted promoter sequence but encode a protein <80% of the intact CPXV homolog from the carboxyl terminus compared to the intact CPXV consensus sequence. ^6^ B29R/C23 is a diploid copy of C23L/B29R (CPXV-GRI stop codon 1393 and is not shown). ^7^ This ORF is not annotated in public sources and is flanked by ORF017 and 018; the stop codons are (24,755 for ECTV, 15,734 for TATV, and 19,312 for VACV-COP.

**Table 2:** Changes in cytokine levels in splenocytes of BALB/c mice infected with TATV and ECTV ^1^.

Target		ECTV				TATV			
Chemokines		Mock	12 H	24 H	48 H	Mock	12 H	24 H	48 H
MIP-2	Chemotactic for polymorphonuclear leukocytes and hematopoietic stem cells	12.2 ± 4.3	2.6 ± 1.8	12.4 ± 2.6	23.5 ± 2.8	8.0 ± 3.8	14 ± 8.1	**182.7 ± 4.4 ^2^**	**260.0 ± 11.4**
MCP-1	Recruits monocytes, memory T cells, and dendritic cells	0.6 ± 0.2	3.5 ± 0.5	3.3 ± 0.1	1.7 ± 0.7	0.5 ± 0.1	0.3 ± 0.2	**11.6 ± 0.1**	**22.9 ± 1.1**
MIP-1β	Activate neutrophils, eosinophils and basophils	27.2 ± 2.3	0.0 ± 0.0 ^3^	0.6 ± 0.3	0.7 ± 0.5	20.7 ± 0.1	23.1 ± 6.6	**64.1 ± 5.4**	**93.5 ± 6.9**
MIP-1α	Activate neutrophils, eosinophils and basophils	22.9 ± 0.1	1.2 ± 0.3	1.4 ± 0.3	1.3 ± 0.2	22.7 ± 2.1	24.2 ± 6.6	**63.6 ± 2.8**	**91.0 ± 5.8**
MIG	Chemoattractant for stimulated T-cell	9.5 ± 0.1	**6.1 ± 0.7**	**15.0 ± 1.5**	**16.1 ± 1.7**	8.6 ± 1.7	7.2 ± 1.6	14.7 ± 2.8	15.1 ± 2.1
RANTES	Chemotatic for T-cells, monocyte lineages, eosinophils, and basophils	8.4 ± 0.4	0.0 ± 0.0	0.0 ± 0.0	0.0 ± 0.0	6.7 ± 0.8	6.8 ± 1.7	**14.7 ± 1.2**	**16.4 ± 0.7**
**Cytokine**									
TNF-α	Systemic inflammation and stimulation of the acute phase reaction	0.2 ± 0.0	0.0 ± 0.0	**1.5 ± 0.2**	**1.8 ± 0.1**	0.0 ± 0.1	0.4 ± 0.3	**1.9 ± 0.2**	**3.4 ± 0.5**
**Growth Factors**									
VEGF	Highly specific mitogen for vascular endothelial cells	0.6 ± 0.2	**27.0 ± 1.1**	**39.8 ± 9.1**	**44.9 ± 9.1**	0.1 ± 0.1	0.6 ± 0.3	**6.3 ± 1.2**	**9.5 ± 1.5**
KC	Chemotaxis and cell activation of neutrophils	2.3 ± 0	2.7 ± 0.1	**9.2 ± 1.2**	**13.5 ± 1.3**	2.4 ± 0.4	2.7 ± 1.5	**30.8 ± 4.1**	**50.2 ± 3.9**
M-CSF	Proliferation and differentiation of macrophages and monocytes	0.4 ± 0.2	**3.7 ± 0.1**	**8.2 ± 2.7**	**7.5 ± 1.0**	0.3 ± 0.2	0.0 ± 0.0	1.0 ± 0.2	**2.3 ± 0.1**

^1^ 1 × 10^6^ splenocytes from BALB/c mice were removed to RPMI-10 and infected with an MOI = 0 (controls) or an MOI = 0.1 of TATV or ECTV. At 12, 24 and 48 h.p.i. supernatants were removed and tested for cytokine levels. Pooled data from 3 experiments. ^2^ Values in bold are increased compared to mock (*p* < 0.05). ^3^ Value underlined are decreased compared to mock (*p* < 0.05).

**Table 3:** Comparison of TNF regulatory genes in TATV, CPXV, CMLV, ECTV, MPXV, VACV, and VARV.

CPXV-GRI/GER Stop	VACV-COP	TNFR	TATV	CPXV-GRI	CPXV-GER91	CMLV-M96	ECTV-MOS	MPXV-ZAR	VACV-WR	VARV-BRA66
14,947		vCD30	Miss ^1^	Gene	Gene	Miss	Gene	Miss	Miss	Miss
213,858		crmD	Miss	Gene	Trnc	Miss	Gene	Miss	Miss	Miss
214,439		crmE	Miss	Gene	Gene	Trnc	Trnc	Miss	Miss	Miss
176,775	A53R	crmC	Trnc ^2^	Gene	Gene	Miss	Miss	Miss	Trnc	Miss
221,381 ^1^	B28R/C22L	crmB	Trnc	Gene	Gene	Gene	Miss	Gene	Trnc	Gene

^1^ Missing (Miss), ^2^ truncated (Trnc), or fragmented (Frag) TNFs are indicated. Full ORFs are indicated as Gene.

## Supplementary Materials

The following are available online at http://www.mdpi.com/1999-4915/10/9/463/s1.
