# Supplementary material for: The Virology of Taterapox Virus In Vitro
Source: Viruses. 2018 Aug 29;10(9):463. doi: 10.3390/v10090463 (PMC6163509; doi:10.3390/v10090463)
Supplement: Supplementary file 1 [file viruses-10-00463-s001.pdf]

Table Supplemental 1. Intact ORFs found in CPXV that are fragmented, truncated or missing in TATV[1].

CPXV-GRI/GER Stop[2, 3]	VACV-COP ortholog	Function (motif)	TATV Status	TATV Protein Size (% compared to CPXV)	CPXV Status	CPXV Protein Size
5392	C17L;C18L/B23R; B24R[4]	Ankyrin; host range protein	Frag[5]		ORF004	672
35634	C2L	Kelch-like	Frag		ORF032	512
38413	N2L	Alpha amanitin sensitivity protein	Frag		ORF035	175
170420	A46R	Interleukin 1 (IL-1) signaling inhibitor	Frag		ORF173	240
180331	A57R	Guanylate kinase	Frag		ORF183	197
191696	B12R	Serine/Threonine kinase	Frag		ORF194	283
7652	C16L/B22R	Unknown	Miss		ORF005	153
8281		Alpha amanitin sensitivity protein	Miss		ORF006	219
9217		Unknown	Miss		ORF007	273
10160		Ankyrin	Miss		ORF008	655
12346		C-type lectin	Miss		Trnc010	96
12699		Kelch-like	Miss		ORF011	521
14342		Unknown (partial homology to TNFR CrmB)	Miss		ORF012	202
14947		Tumor necrosis factor receptor (vCD30)	Miss		ORF013	111
15374		Ankyrin	Miss		ORF014	764
18018		Ankyrin	Miss		ORF015	437
19440		Unknown	Miss		ORF016	178
32306		Unknown	Miss		ORF028	29
165942	A40R	Lectin homolog	Miss		ORF166	165
212883	C15L/B21R	Unknown	Miss		ORF206	581
213858		Tumor necrosis factor receptor (CrmD)	Miss		ORF207	322
214439		Tumor necrosis factor alpha receptor (CrmE)	Miss		ORF208	167
215231		N-methly D-aspartate receptor-like protein	Miss		ORF209	210
216015	B22R/C16L	Unknown	Miss		ORF210	153
20088		Ankyrin	Trnc[6]	285 (34)	ORF017	833
22682		Unknown	Trnc	113 (66)	ORF018	170
23924	C10L	Interleukin 1 (IL-1) receptor antagonist	Trnc	27 (8)	ORF020	331
31175	C8L	Unknown	Trnc	74 (41)	ORF026	182

33086	C5L	Unknown	Trnc	150 (73)	ORF029	205
34788	C3L	Complement binding protein (secreted)	Trnc	194 (75)	ORF013	259
37242	C1L	Unknown	Trnc	72 (34)	ORF033	212
41155	K1L	Ankyrin and NFkB inhibitor	Trnc	88 (31)	ORF038	284
50154	F5L	Membrane protein (36 kDa)	Trnc	193 (60)	ORF048	323
73579	O1L	Unknown	Trnc	64 (10)	ORF073	666
150930	A25L	A-type inclusion protein	Trnc	736 (58)	ORF149	1279
163365		Unknown	Trnc	50 (78)	ORF163	64
165419	A39R	Semaphorin	Trnc	134 (33)	ORF165	402
168244	A44L	Hydroxysteroid dehydrogenase	Trnc	88 (25)	ORF171	346
169708	A45R	Superoxide dismutase (Cu-Zn) like protein	Trnc	46 (37)	ORF172	125
171963	A48R	Thymidylate kinase	Trnc	58 (28)	ORF175	204
172501	A49R	Phosphotransferase anion transport protein (putative)	Trnc	77 (48)	ORF176	162
175891	A52R	Intracellular TLR and IL-1 signaling inhibitor	Trnc	51 (27)	ORF179	190
176775	A53R	Tumor necrosis factor receptor (CrmC)	Trnc	47 (25)	ORF180	186
178726	A55R	Kelch-like	Trnc	219 (39)	ORF181	564
190433	B10R	Kelch-like	Trnc	36 (7)	ORF192	501
197359	B18R	N-term ankyrin, C-term F-box	Trnc	147 (26)	ORF199	574
200936	B20R	Ankyrin	Trnc	29 (4)	ORF201	795
221381	B28R/C22L[7]	Tumor necrosis factor (TNF) receptor (CrmB)	Trnc	98 (28)	ORF213	351
222274	B29R/C23[8]	Chemokine binding protein	Trnc	61 (24)	ORF214	255

[1]All intact, missing (Miss), truncated (Trnc) or fragmented (Frag) ORFs common between TATV and CPXV are removed. All truncated CPXV ORFs that are full length ORFs in TATV are removed. ORFs are numbered according to PBR number.

[2]The stop codon is used for ORF identification as there sometimes can be multiple in frame ATG's located proximal to the 5' end of the transcript, therefore making the precise start codon for the protein difficult to identify.

[3]Orthopoxvirus central conserved region exists between CPXV-GRI ORF Host Range Virulence Factor (stop codon 31795, VACV-COP C7L) and an ORF of unknown function (stop codon 175249, VACV-COP A51R) which corresponds to approximately 15.3 - 157.3 kbp in TATV and 15.3 - 157.3 kbp in CPXV.

[4]Diploid copy not shown.

[5]Any ORF that has lost its predicted promoter and/or has been significantly truncated at its 5' end is annotated as a fragmented gene.

[6]ORFs that maintain a predicted promoter sequence, but encode a protein <80% of the intact CPXV homolog from the carboxyl terminus sequence.

[7]B28R/C22L is a diploid copy of C22L/B28R (CPXV-GRI stop codon 2286 and is not shown).

[8]B29R/C23 is a diploid copy of C23L/B29R (CPXV-GRI stop codon 1393 and is not shown).

Table Supplemental 2. Intact ORFs found in VARV that are fragmented, missing or truncated in TATV[1].

CPXV-GRI/GER Stop[2, 3]	VACV-COP ortholog (CPXV protein size)	Function (motif)	TATV Status	TATV Protein Size (% compared to CPXV)	VARV Status	VARV Protein Size (% compared to CPXV)
7652	C16L/B22R (153)	Unknown	Miss		ORF001	153 (100)
32306	(29)	Unknown	Miss		ORF012	29 (100)
38413	N2L (175)	Alpha amanitin sensitivity protein	Frag[4]		ORF022	177 (99)
170420	A46R (240)	Interleukin 1 (IL-1) signaling inhibitor	Frag		ORF165	240 (100)
23924	C10L (331)	Interleukin 1 (IL-1) receptor antagonist	Trnc[5]	27 (8)	ORF016	330 (100)
34788	C3L (259)	Complement binding protein (secreted)	Trnc	194 (75)	ORF017	263 (102)
37242	C1L (212)	Unknown	Trnc	72 (34)	ORF020	214 (101)
50154	F5L (323)	Membrane protein (36 kDa)	Trnc	193 (60)	ORF035	322 (100)
73579	O1L (666)	Unknown	Trnc	64 (10)	ORF060	666 (100)
163365	(64)	Unknown	Trnc	50 (78)	ORF151	62 (97)
169708	A45R (125)	Superoxide dismutase (Cu-Zn) like protein	Trnc	46 (37)	ORF164	125 (100)
171963	A48R (204)	Thymidylate kinase	Trnc	58 (28)	ORF167	205 (100)
172501	A49R (162)	Phosphotransferase anion transport protein (putative)	Trnc	77 (47)	ORF168	162 (100)
197359	B18R (574)	Unknown (Ankyrin)	Trnc	147 (26)	ORF181	574 (100)
200936	B20R (795)	Unknown (Ankyrin)	Trnc	29 (4)	ORF198	786 (100)
221381	B28R/C22L[6] (351)	Tumor necrosis factor (TNF) receptor (CrmB)	Trnc	98 (28)	ORF206	349 (99)
222274	B29R/C23[7] (255)	Chemokine binding protein	Trnc	61 (24)	ORF207	253 (99)

[1]All intact, missing (Miss), truncated (Trnc) or fragmented (Frag) ORFs common between TATV and VARV are removed.

All truncated VARV ORFs that are full length ORFs in TATV are removed.

[2]The stop codon is used for ORF identification as there sometimes can be multiple in frame ATG's located proximal to the 5' end of the transcript, therefore making the precise start codon for the protein difficult to identify.

[3]Orthopoxvirus central conserved region exists between CPXV-GRI ORF Host Range Virulence Factor (stop codon 31795,VACV-COP C7L) and an ORF of unknown function (stop codon 175249, VACV-COP A51R) which corresponds to approximately 15.3 - 157.3 kbp in TATV and 9.1 - 150.1 kbp in VARV.

[4]Any ORF that has lost its predicted promoter and/or has been significantly truncated at its 5' end is annotated as a fragmented gene.

[5]ORFs that maintain a predicted promoter sequence but encode a protein <80% of the intact CPXV homolog from the carboxyl terminus CPXV consensus sequence.

[6]B28R/C22L is a diploid copy of C22L/B28R (CPXV-GRI stop codon 2286 and is not shown).

[7]Haploid gene in VARV (diploid copy missing).
